# First evaluation of the population structure, genetic diversity and landscape connectivity of the Endangered Arabian tahr

**DOI:** 10.1007/s42991-020-00072-4

**Published:** 2020-10-13

**Authors:** Steven Ross, Jean-Marc Costanzi, Mansoor Al Jahdhami, Haitham Al Rawahi, Muhammad Ghazali, Helen Senn

**Affiliations:** 1 Office for Conservation of the Environment, Diwan of Royal Court, P.O. Box 246, 100 Muscat, Sultanate of Oman; 2 WildGenes Laboratory, Conservation Department, Royal Zoological Society of Scotland, Edinburgh, EH12 6TS UK

**Keywords:** Arabian tahr, Hajar mountains, Genetic diversity, Genetic structure, Connectivity, Corridors

## Abstract

The Arabian tahr (*Arabitragus jayakari*) occurs only in the mountains of northern Oman and the United Arab Emirates. The species is classified as Endangered due to its small declining population. In this study, we combined genetic and landscape ecology techniques in order to inform landscape scale conservation and genetic management of Arabian tahr. Using 540 base pairs of mitochondrial control region in a dataset of 53 samples, we found eight haplotypes, which fell into two haplogroups. Population genetic analysis using a panel of 14 microsatellite loci also showed a weak, but significant division. Analyses of landscape connectivity supported the genetic results showing poor connectivity between populations in the far south of the study area and those in the north. The most likely location of corridors connecting Arabian tahr populations were identified. Many corridors between tahr populations are impeded by multi-lane highways and restoration of these connections is required to maintain population viability of Arabian tahr. Owing to limited genetic samples outside of Wadi Sareen, further sampling is needed to elucidate both mtDNA and the nuclear structure of Arabian tahr more fully. Our study provides a toolkit that may be used for future genetic and connectivity monitoring of the Arabian tahr population.

**Electronic supplementary material:**

The online version of this article (10.1007/s42991-020-00072-4) contains supplementary material, which is available to authorized users.

## Introduction

Anthropogenic habitat loss and fragmentation is known to negatively affect biodiversity by reducing suitable habitat area, habitat quality and connectivity between populations, and is recognised as the greatest threat to global biodiversity (Sala et al. 2000; Fahrig 2003; Baden et al. 2019). Because habitat loss and fragmentation often block natural connections between populations, conservationists have advocated the retention of habitat corridors, which are widely regarded as essential for the persistence and viability of wildlife populations in fragmented landscapes (Beier and Noss 1998). Corridors facilitate movement and connectivity between isolated habitat patches. Although individual patches may be too small to maintain populations of area sensitive species, a well-connected network of habitat patches can provide sufficient area to maintain viable populations (Noss 2004).

Over the long-term, reduced connectivity results in increased extinction risk, particularly for Endangered species with small populations (Frankham 2015). As population size decreases, allelic variants found within the total complement of genes, *the genepool*, drift to extinction resulting in an erosion of genetic diversity. Loss of genetic diversity results in reduced evolutionary flexibility and inability to adapt to future threats such as disease and climate change (Reed and Frankham 2003; Frankham et al. 2014). In the case of threatened species with small fragmented populations, such as the Arabian tahr (*Arabitragus jayakari*), genetic management should be considered an important part of conservation management.

**Fig. 1:**
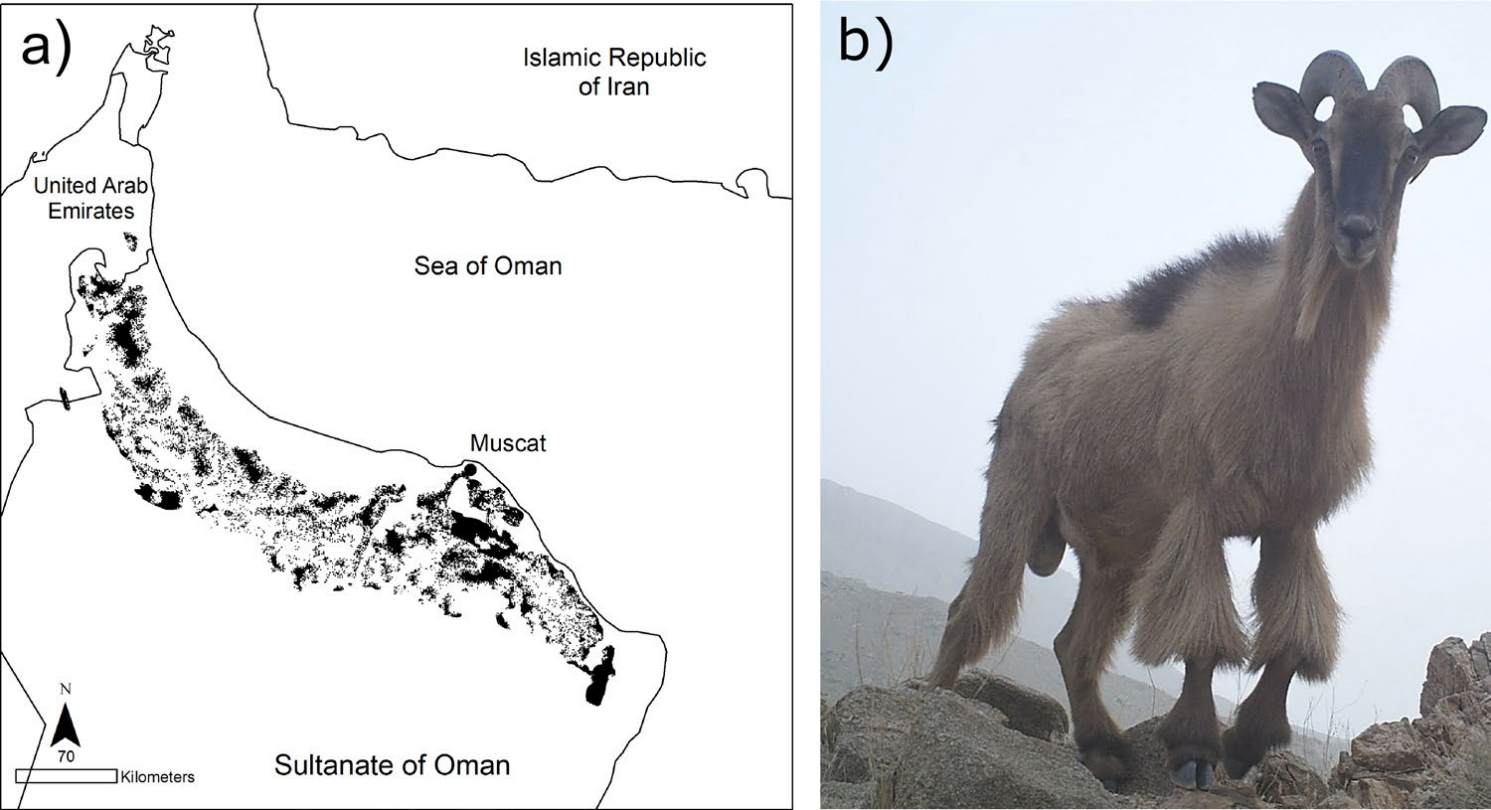
**a** The world distribution of Arabian tahr indicated by area of occupancy (black shading). **b** A photograph of a male Arabian tahr photographed in Oman

The Arabian tahr is an Endangered caprid endemic to the Hajar Mountains (Ross et al. 2019), spanning a 600 km mountainous crescent in northern Oman and the eastern United Arab Emirates (Fig. 1). The species is little known largely due to its cryptic nature and the difficulty of working in its preferred steep, rocky habitat (Ross et al. 2017). Aside from a limited phylogenetic analyses of the species (Ropiquet and Hassanin 2005), no genetic study of the Arabian tahr has been published to date. The Arabian tahr is listed as Endangered by the IUCN due to its small declining population being subject to a number of threats, including habitat loss and fragmentation, competition with livestock and poaching (Ross et al. 2019). Within the mountains, the species prefers steep, rugged cliff habitats at elevations between 400 and 1200 m and is rarely found in flat terrain without adequate cover, although it is known to disperse through such habitat (Ross et al. 2017). The most important Arabian tahr populations occur in Wadi Sareen and Jabal Qahwan Nature Reserves, but many small populations still survive outside of the protected area network (Ross et al. 2017), and these populations are likely to be important for the maintenance of connectivity and gene flow.

Owing to the relevance of both connectivity and conservation genetics to the Endangered Arabian tahr, we combined genetic and landscape ecology techniques to provide a genetic toolkit and recommendations for landscape scale conservation of Arabian tahr. Our specific objectives were to (1) provide a conservation genetic toolkit for Arabian tahr to facilitate future assessment and monitoring of the species; (2) evaluate genetic diversity of remaining Arabian tahr populations; (3) identify population genetic structure; (4) assess landscape connectivity and how it may impact Arabian tahr gene flow and population persistence; and (5) provide management recommendations to maintain population connectivity.

## Methods

### Data collection

We searched for genetic samples of the Arabian tahr while deploying camera traps at 336 locations across the Hajar Mountains of Oman and while trapping Arabian tahr at three locations in Wadi Sareen Natural Park, between September 2011 and June 2015. Sixty-seven genetic samples were collected over seven areas of the south-eastern Hajar Mountains (Table 1). Despite similar effort in the northern Hajar Mountains no samples were found in the northern distribution. Samples were of two types: firstly EDTA Blood and ear tissue punches stored in 80% ethanol which were collected directly from animals trapped and GPS-collared for the study in Wadi Sareen, and secondly faecal pellets, and desiccated tissue or bone samples from tahr carcasses, collected during camera trapping surveys and stored along with a silica drying agent (Table 1).

**Table 1:** Breakdown of the samples collected for the study

Location name	*n*	Number of non-invasive samples (Carcass and faecal pellets)	Samples sequenced successfully (mtDNA)	Samples genotyped successfully (microsatellite)	Samples included in the ddRAD analysis	Samples failed to sequence	Samples sequenced but not tahr
Wadi Sareen	42	17	39	28	21 (19 successful)	3 (all carcass)	0
Ras Ash Shajar	3	3	2	1	0	0	1 (sheep, pellet)
Tiwi	1	1	1	1	0	0	0
As Saleel	11	11	5	2	0	6 (all pellets)	1 (sheep, pellet)
Jabal Qahwan	8	8	6	5	0	0	0
Al Khabura Cam	1	1	0	0	0	1 (pellet)	0
Samail	1	1	0	0	0	0	1 (domestic goat, carcass)

### DNA extraction

DNA extraction from blood and tissue samples was conducted using the following protocols FujiFilm Tissue/Blood Kit (Raytek Scientific Ltd.). For faecal samples, a combination of Isohelix DNA Isolation Kit (Cell Projects Ltd.) and QIAamp DNA Stool Mini Kit (Qiagen Ltd.) were used for DNA extraction following the protocol described in Werhahn et al. (2017). In order to decrease the risk of contamination for non-invasive samples, all extractions were conducted in a fume cupboard equipped with UV light. Between each extraction batches the fume cupboard was cleaned and exposed to UV light. When preparing the samples before extraction gloves were changed and the equipment was cleaned and sterilised between each sample.

### mtDNA sequencing

For each sample, 540 base pairs of mitochondrial control region (d-loop) was amplified with the following primers TAH D-loop1 forward (5′-GAAGTTCTACTTAAACTATTCCCTG-3′) and primers TAH D-loop1 reverse (5′-AGAAAGAACCAGATGTCTGATA-3′) according to the following conditions, 95 °C for 5 min; 40 cycles of 95 °C for 30 s, 58 °C for 30 s and 72 °C for 1 min; 72 °C for 10 min.

For samples where d-loop sequences were not obtainable, universal mammalian cytochrome B primers (Verma and Singh 2003) were used to verify that lack of amplification was not due to incorrect species status of the sample using the conditions 95 °C for 5 min; 40 cycles of 95 °C for 30 s, 50 °C for 30 s and 72 °C for 1 min; 72 °C for 10 min.

PCR reactions were prepared in a total volume of 10 μl using 1 μl DNA, 7 μl 2X Maxima Hot Start PCR Master Mix (ThermoFisher Scientific Ltd.) and 1 μl of 10 nM of each primer. PCR product was then purified using 1 μl mix of Exonuclease I (ThermoFisher Scientific Ltd.) and FastAP Thermosensitive Alkaline Phosphatase (ThermoFisher Scientific Ltd.) with 1:1 ratio. All products were sequenced in the forward direction (a subset of unique haplotypes were also done in the reverse direction) using a BigDye Terminator v3.1 Cycle Sequencing Kit (ThermoFisher Scientific Ltd.) with PCR conditions: 96 °C for 1 min; 25 cycles of 96 °C for 10 s, 50 °C for 5 s and 60 °C for 4 min; 4 °C for 10 min. For every reaction a negative control was included and analysed in parallel with the samples. If any positive signal was found in the negative control the results were rejected and the entire run was repeated.

The mitochondrial DNA sequences were edited with the software Geneious 8.1.6. (https://www.geneious.com).

### Microsatellite analysis

The following 32 microsatellite primers were screened for polymorphism: 11HDZ550, BM1329, BM302, BM3224, BM4505, BM4513, BM848, BMS4008, CSSM043, D5S2, ETH10, ETH225, HEL1, INRA005, INRA107, INRA40, MAF46, MAF50, MAF70, MCM38, OarAE119, OarCP26, OarFCB304, RBP3, RM088, RT5, RT6, SPS113, SR-CRSP6, TEXAN6, TGLA122 and TGLA94 using a set of 7 Arabian tahr samples (plus a negative control) (for details and references see Online Appendix 1). After markers elimination for monomorphism, poor scoring or non-amplification was applied, 15 markers were then selected for the final three multiplex panels (see Online Appendix 2).

The microsatellite panels were run under the following PCR conditions: 95 °C for 5 min; 40 cycles of 95 °C for 30 s, 50 °C for 30 s and 72 °C for 1 min; 72 °C for 10 min. Total 10 μl reaction was performed for PCR amplification: 1 μl template DNA, 5 μl Type-it and 1 μl Q-solution (Qiagen, Ltd.), 2 μl of ddH_2_O and 0.5 μl of each forward and reverse primer. As for the mtDNA sequencing, for every PCR reaction a negative control was included and if a positive signal was observed the entire run was repeated. To confirm PCR amplification, the products were then visualised on a 1.5% agarose gel. For non-invasive samples (faecal, tissue) each sample was repeated three times. Consensus calls were generated from three identical homozygous profiles or two identical heterozygous protocols and ambiguous profiles were scored another two times and the consensus taken. We used Geneious version 8.1.6 to score the 15 microsatellites loci.

Microsatellite loci were tested for the presence of null alleles with the software micro-checker (Van Oosterhout et al. 2004). Hardy–Weinberg equilibrium and linkage disequilibrium were calculated with the software GENEPOP V4.3 (Raymond and Rousset 1995) and significance levels were adjusted with Bonferroni correction. Finally, the probability of identity (PID) and probability of identity for siblings (PIDsibs) were estimated with the Genalex 6.5 (Peakall and Smouse 2012).

### ddRAD analysis

#### ddRAD wet lab analysis

DNA extracts from blood and ear tissue samples from Wadi Sareen were assessed for DNA quality via agarose gel electrophoresis on a 2% gel, and 21 samples (TAH044-55, TAH057-59 and TAH060-64) with suitable non-degraded DNA selected for the library preparation stage. DNA was quantified using a Qubit Assay (Thermofisher Scientific) and normalised to 7 ng–1 µl.

A double digest RAD (ddRAD) library was produced according to a modified protocol of Peterson et al. (2012) (see also Bourgeois et al. (2018) for the modified protocol) using the restriction enzymes SphI and SbfI and a gel excision section of 320–590 bp following restriction of the DNA with the two enzymes. Individuals in the library were barcoded using a barcode on each end of the fragment (double barcode combination) and each individual was repeated twice with a different barcode across the library to improve evenness of coverage. The barcoding system allows DNA from multiple individuals to be “tagged” and pooled into a single lane of sequencing. An additional positive control was included to allow for quality control of the experimental process and for assessment of genotyping error-by-read depth. The resulting library was quantified using a Qubit Assay (Thermofisher Scientific) and then sequenced in both directions (Read 1 and Read 2) using a single lane of MiSeq Sequencing Technology (Illumina).

#### ddRAD bioinformatics

The sequences were quality assessed using FastQC (https://www.bioinformatics.babraham.ac.uk/projects/fastqc/) and the reads were then demultiplexed by barcode using the *process_radtags* module of the Stacks 1.44 bioinformatics pipeline (Catchen et al. 2013). The reads were simultaneously quality filtered “cleaned” using the same module and low quality reads discarded. Demultiplexed read files were then concatenated into read files for each individual (four barcode combinations per individual, see above) and for each individual the read 1 and 2 files were concatenated into a single file.

The individual data was then processed using the *denovo_map.pl* module of Stacks (-m 3 -M 3 -n 0) to assemble and create a catalogue of genetic loci contained in the data. The Stacks scripts *export_sql.pl* (snps_l = 1 -F snps_u = 1 -F alle_l = 2 -F alle_u = 2) and Stacks scripts *populations* (-m 10) and Plink v1.07 toolset (Purcell et al. 2007) were then used to filter all loci that fulfilled the following criteria:

1. Contained exactly 1 SNP to facilitate the design of high quality primers if desired later on and remove non-independent markers;
2. Contained exactly two alleles, as the presence of more than two alleles might represent genome duplication;
3. Had a read depth of ≥ 10 ten reads per individual to maximise the likelihood of the SNP being true.
4. Had genotyped in ≥ 18/21 of the Arabian tahr samples.

### Population genetic analysis

Haplotype analysis and haplotype AMOVA were conducted in PopArt version 1.7 (Leigh and Bryant 2015).

Basic population genetic exploration and quality control of the microsatellite data, including searching for duplicate profiles, was conducted in Genalex version 6.5 (Peakall and Smouse 2012). The AMOVA calculation for the microsatellite dataset was also calculated with Genalex version 6.5 (Peakall and Smouse 2012). The expected heterozygosity (*H*_e_), observed heterozygosity (*H*_o_) and inbreeding coefficient (*F*_is_) were calculated for the genetic cluster defined by the STRUCTURE results with the software Genetix v4.05.2 (Belkhir et al. 2004). Pairwise *F*_st_ and significance probabilities were obtained with the software Arlequin (Ver 3.5) (Excoffier and Lischer 2010).

Principle component analysis (PCA) and estimation of the number of genetic cluster (*K*) of the microsatellite and SNP data were conducted in the adegenet package of R (1.3-1) (Jombart 2008; Jombart and Ahmed 2011). Bayesian estimation of population structure were inferred with the software STRUCTURE 2.3.4 (Pritchard et al. 2000) using the admixture and correlated allele frequencies models with a burn in of 1,000,000 followed by 2,000,000 MCMC iterations. The LOCPRIOR option (the sample location was used as a prior), was also enabled as it tends to perform better than models without LOCPRIOR enabled (Hubisz et al. 2009). For each analysed dataset, ten replicate runs were performed for each value of the number of clusters (*K*) from one to six. The CLUMPAK web interface was used to summarise the results and graphs (Kopelman et al. 2015). The most likely number of clusters (*K*) was determined using the calculation of delta *K* (Δ*K*) described in Evanno et al. (2005), as implemented by the web interface of STRUCTURE HARVESTER (Earl and vonHoldt 2012), but also visually directly from STRUCTURE’s bar plots. STRUCTURE was also run for the SNP dataset with the same configuration than above but for *K* 1–3 and without the LOCPRIOR (as the individuals all came from the same area). In order to support the results from STRUCTURE we used the software GENELAND to create a spatially explicit model for the inference of the number of genetic cluster (*K*) and the spatial location of genetic discontinuities between those clusters. We ran the software for 1–6 clusters (*K*) using 1,000,000 iterations and a thinning of 100. The allele frequency model was set to correlated and ten independent runs were realised. The rest of the parameters were left to the default.

### Landscape connectivity

To investigate Arabian tahr connectivity and quantify potential movement corridors across its south-eastern range we used Circuitscape (McRae et al. 2013a) and Linkage Mapper (McRae and Kavanagh 2011) toolkits implemented within ArcMap 10.3.1 (ESRI 2015) software. In Circuitscape, connectivity is based on circuit theory, which predicts the most probable movement patterns of random walkers between source populations and target areas based on resistance to movement. Output maps show current flow, where high current flow indicates the most probable and important areas for movement between habitat patches (Mcrae et al. 2008). Linkage Mapper uses vector maps of core habitat areas and raster maps of resistance to movement to identify and map least-cost linkages between core areas (McRae and Kavanagh 2011). We used Circuitscape to create a map of areas facilitating movement between core habitats and Linkage mapper to identify the most likely corridors to be used by Arabian tahr.

The database used to create the resistance surface for Circuitscape included an Arabian tahr distribution model, surface ruggedness, roads and villages. The distribution model was created using a single season multi-variate occupancy model based on 300 camera traps systematically set across the Arabian tahrs’ range (Ross et al. 2017), allowing incorporation of habitat suitability into analyses, where more suitable habitats were given lower resistance to movement. Surface ruggedness, which is a measure of surface roughness and terrain complexity, was derived from a digital elevation model and used to indicate anti-predator habitat of Arabian tahr, which is a known requirement of the species (Ross et al. 2017). Roads and villages were used to indicate impediments to movement. Roads were split into highways which were multilane roads and other surfaced roads that had single lane traffic. Highways and villages were given the highest resistance values as tahr generally avoid these areas (Ross et al. 2017).

Circuitscape and Linkage Mapper model parameters were taken from the GIS database of the study area. A landscape resistance surface at 250 m pixel resolution was constructed using Gnarly Landscape Utilities (McRae et al. 2013b). A tahr habitat distribution model and anthropogenic variables were assigned resistance values according to the ability of Arabian tahr to use and cross habitats. Insight into parameterisation of resistance was gained from a tahr occupancy study (Ross et al. 2017) and unpublished GPS collar and habitat selection data for 26 Arabian tahr (Ross et al. in Prep).

The Arabian tahr distribution model was also used to indicate core tahr populations which acted as current sources in Circuitscape and were given a value of zero resistance. Core populations were defined as pixels where tahr occupancy probability was greater than 0.6 and in clusters of greater or equal to 40 pixels (using an 8-cell neighborhood). Although tahr are known to occupy areas of lower occupancy probability, our criteria ensured that enough tahr inhabited the core areas to act as breeding and dispersing source populations from where current could be inputted into the connectivity circuit (McRae et al. 2013a). Circuitscape was used in pairwise mode to calculate current flow across all possible pairs of core tahr populations and summed to produce maps of cumulative current density. Linkage Mapper was used to map the least cost paths (LCPs) to estimate the minimum cost-weighted distance between adjacent core tahr populations (Adriaensen et al. 2003).

Five connectivity models were run in Circuitscape, to understand the impact of varying resistance values of variables. This included one theorical model without highways and with more favorable habitat conditions for movement, to mimic a landscape prior to the 1990s when human population sizes and anthropogenic impacts were much lower. The best connectivity model was selected using the area under the curve value (AUC) of a receiver operating characteristic curve (ROC; Pearce and Ferrier 2000), using an online ROC application (Eng 2014). We used a dataset of 94 camera traps to validate connectivity models. All cameras were located outside of core tahr populations so that the test was focused on the quality of linkages. We tested the predicted current (amps) of camera trap sites ability to discriminate between cameras that detected or failed to detect Arabian tahr (1/0).

The best cumulative current model and corridors were mapped to represent connectivity of Arabian tahr across the study area. Corridors between populations were represented using the ratio of cost-weighted distance (CWD) to the path length (PL). The ratio indicated the average resistance an individual experiences when using the optimal path to move between populations (e.g. Dutta et al. 2016). All corridors over a CWD/PL ratio of 2.0 were cropped to provide realistic corridors that could be used to suggest management actions.

## Results

### mtDNA data

In total 53 samples generated 540 base pairs of mitochondrial control region (d-loop). Three failed samples were found not to be Arabian tahr following sequencing at cytochrome B (Table 1). These were discarded from further analysis.

**Fig. 2:**
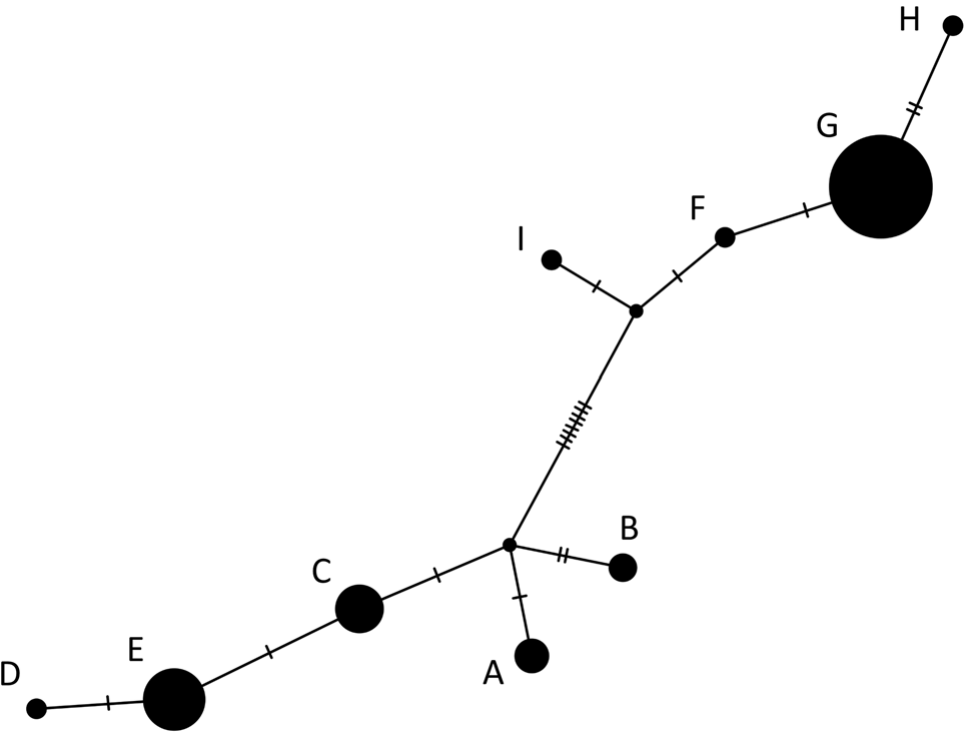
Haplotype network of 540 base pairs of mitochondrial control region (d-loop) in Arabian tahr. Each hatch marks represents a one base pair mutation. The geographic provenance of each haplotype (A–H) can be found in Fig. 3. Note that the haplotype I correspond to the haplotype of the individual from the United Arab Emirates

The mitochondrial control region sequence data consisted of 8 haplotypes which were named A–H and deposited on GenBank under the accession numbers MN563184—MN563191. One further haplotype from an animal from the United Arab Emirates was included from the NCBI GenBank accession FJ207523 (Hassanin et al. 2009). This haplotype was renamed “I” (Fig. 2). Further details as to this individual’s geographical origin were unavailable.

In total there were 19 segregating sites between the haplotypes and 12 parsimony informative sites. Nucleotide diversity was 0.011. Both minimum-spanning and median-joining haplotype networks revealed stable arrangement with two main haplogroups 1 (A,B,C,D,E) and 2 (F,G,H,I) divided by 10 stepwise mutations. Within each haplogroup, haplotypes were divided by maximally 3 step-wise mutations from their nearest neighbour (Fig. 2, see also Online Appendix 3 for details on the position of each SNP).

**Fig. 3:**
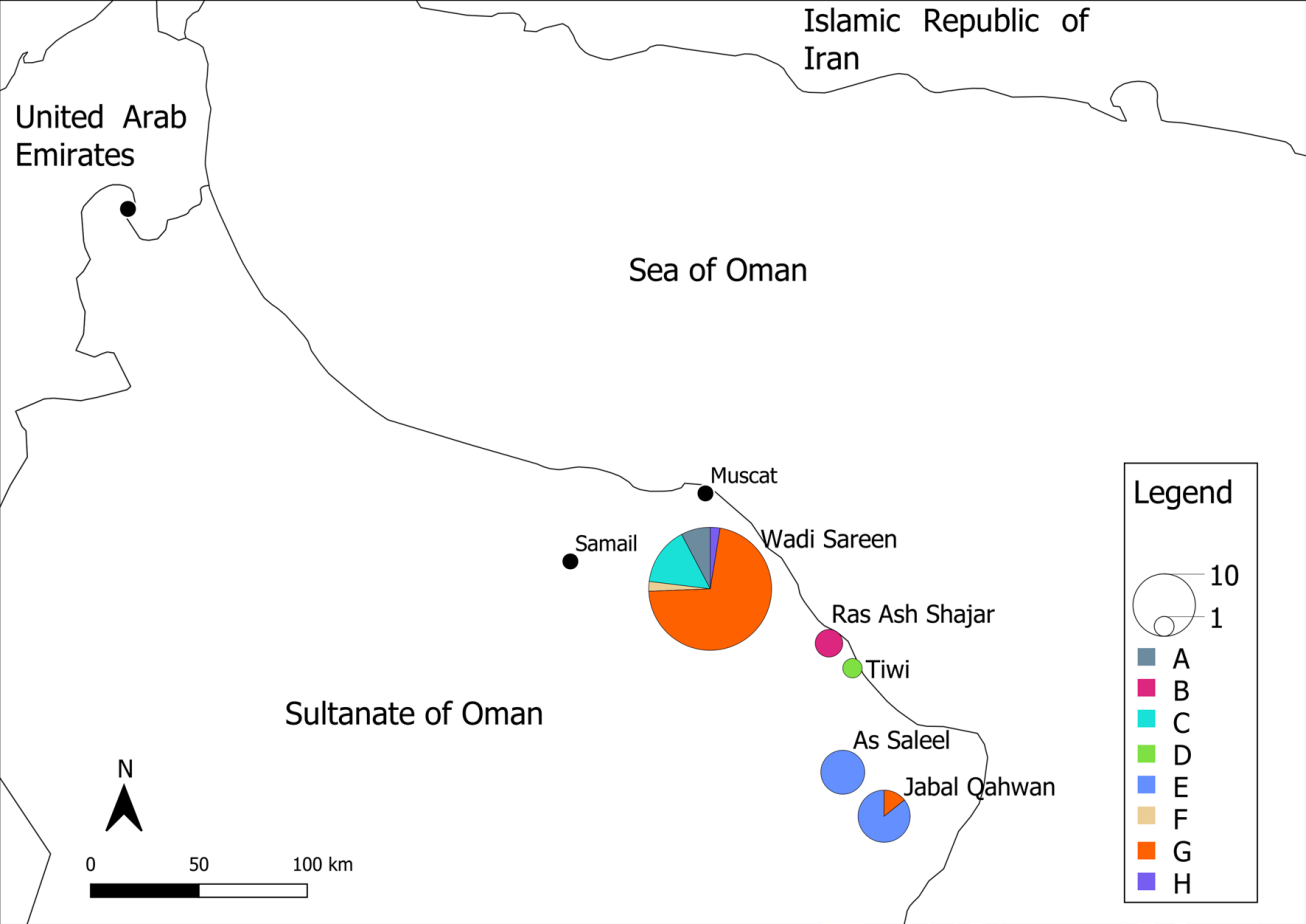
Mapped location of haplotypes in the location samples were collected and their respective name. The black dots represent major cities in the area and the black lines regions and country borders. The United Arab Emirates haplotype has been placed in Al Ain because there was no associated geographical location given

Geographical segregation of the haplotypes appeared to be fairly strong with recovered haplotypes being restricted to a single sample area, apart from haplotype E found in Jabal Qahwan and As Saleel and Haplotype G found in Wadi Sareen and Jabal Qahwan (Fig. 3). The most sampled site, Wadi Sareen, contained haplotypes from both haplogroups, whereas samples gathered from south of Wadi Sareen predominantly featured haplotypes from the haplogroup 2. The analysis of molecular variance (AMOVA) is summarised in Table 2, and highlights that the majority of variance in haplotype frequency was attributable to the separation between Wadi Sareen and the southern populations (67.3% of the variation; permutation test *P* < 0.001). However, a significant proportion of the variance was also attributable to the difference among samples within populations (36.2%; permutation test *P* < 0.001). The difference between populations within the southern haplogroup was not significant (which may potentially be a function of small sample size).

**Table 2:** AMOVA at mtDNA d-loop for Arabian tahr samples from Oman grouped into “Wadi Sareen” and “south of Wadi Sareen”. The United Arab Emirates haplotype was ignored in this analysis

Variation	*df*	Sum of squares	Variance components	%Variation
Among groups (*Wadi Sareen* versus south of *Wadi Sareen*)	1	768.107	37.592	67.299
Among populations within groups	3	42.643	− 1.934	− 3.462
Within populations	48	969.590	20.200	36.163
Total	52	1780.340	55.858	

### Microsatellite data

In total, 37 samples were successfully genotyped (Table 1). Out of the 15 microsatellites selected for the analysis one locus, BM451, had a high frequency of null alleles and was therefore removed from the analysis. All 14 remaining loci were found to be at Hardy–Weinberg equilibrium and no linkage disequilibrium was found among loci after Bonferroni correction. Polymorphism in the 14 microsatellite used for the analysis ranged from 2 (ETH255)—8 (BM4505) alleles per locus. Expected heterozygosity (*H*_e_) ranged from 0.18 (BM4008) to 0.0.77 (MCM38) and observed heterozygosity (*H*_o_) ranged from 0.19 (BM4008) to 0.78 (MCM38) (Table 3).

**Table 3:** Diversity indices for the 14 microsatellite loci studied (*n* = 37)

	OarCP26	BM302	BM4505	ETH10	BM4008	ETH225	INRA40	HEL1	MCM38	BM1329	INRA005	11HDZ550	RT6	MAF50
Na	4	4	8	3	3	2	5	4	7	5	3	5	3	5
*H*_o_	0.54	0.38	0.57	0.38	0.19	0.34	0.43	0.57	0.78	0.63	0.50	0.54	0.44	0.51
*H*_e_	0.59	0.38	0.59	0.52	0.18	0.32	0.68	0.58	0.77	0.66	0.47	0.64	0.53	0.65

*Na* number of alleles, *H*_*o*_ observed heterozygosity, *H*_*e*_ expected heterozygosity

Multi-locus genotype matches were assessed, but there were no genotypes that matched or showed one genotype mismatch. There were three pairs of animals which had identical genotypes at all but two mismatching loci (TAH051 and TAH060, TAH060 and TAH067) which could have potentially been accounted for by allelic dropout; however, these animals were blood-sampled individuals that were given individual identification marks following blood sampling, so accidental resampling of these animals can be ruled out and it can be taken that the genotypes are from two different animals.

The probability of identity (PID) and probability of identity for siblings (PIDsibs) were respectively 3.8 × 10^–8^ and 3.8 × 10^–4^ for Wadi Sareen and respectively 5.0 × 10^–8^ and 5.4 × 10^–4^ for the populations south of Wadi Sareen.

**Fig. 4:**
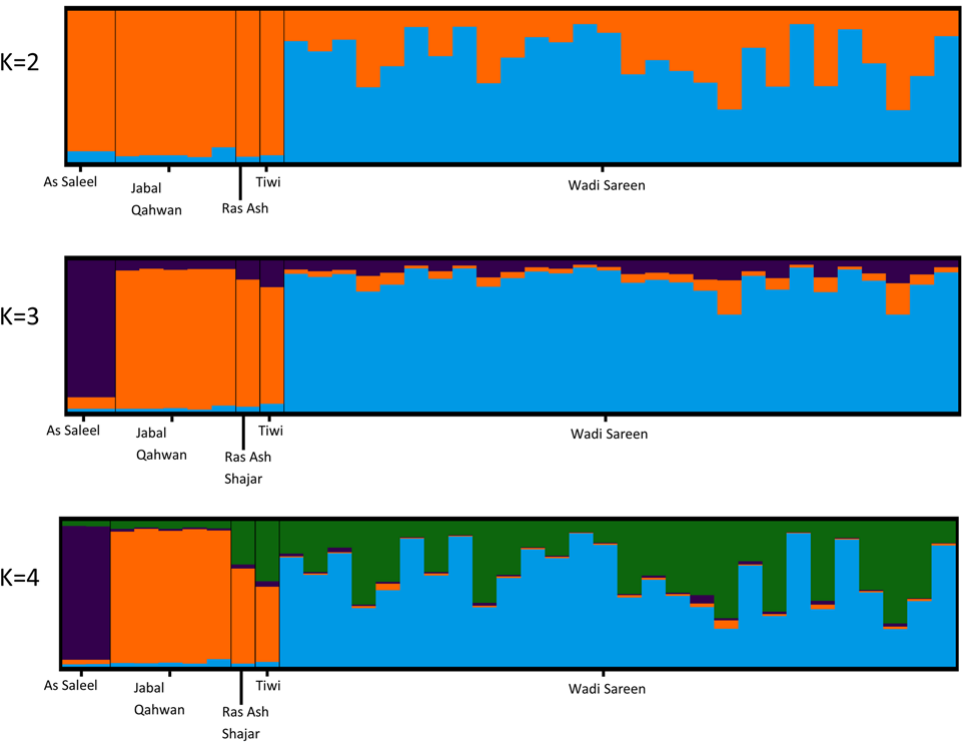
Bar plot presenting the results from STRUCTURE for different numbers of clusters (*K*), the graph was generated with the CLUMPAK web interface (Kopelman et al. 2015)

Analysis of the microsatellite data using STRUCTURE at ten replicates of 1–6 K revealed that the most likely value of *K* was 2 (Δ*K*2 = 14.75) while Δ*K* was Δ*K*3 = 0.83 (see also Online Appendix 4). On the other hand the STRUCTURE bar plot graphs (Fig. 4) indicate further substructure within the southern genetic cluster and a certain level of admixture for the Wadi Sareen genetic cluster. Finally, when the STRUCTURE analysis was run separately for each cluster no clear substructure was observed in both cases.

**Fig. 5:**
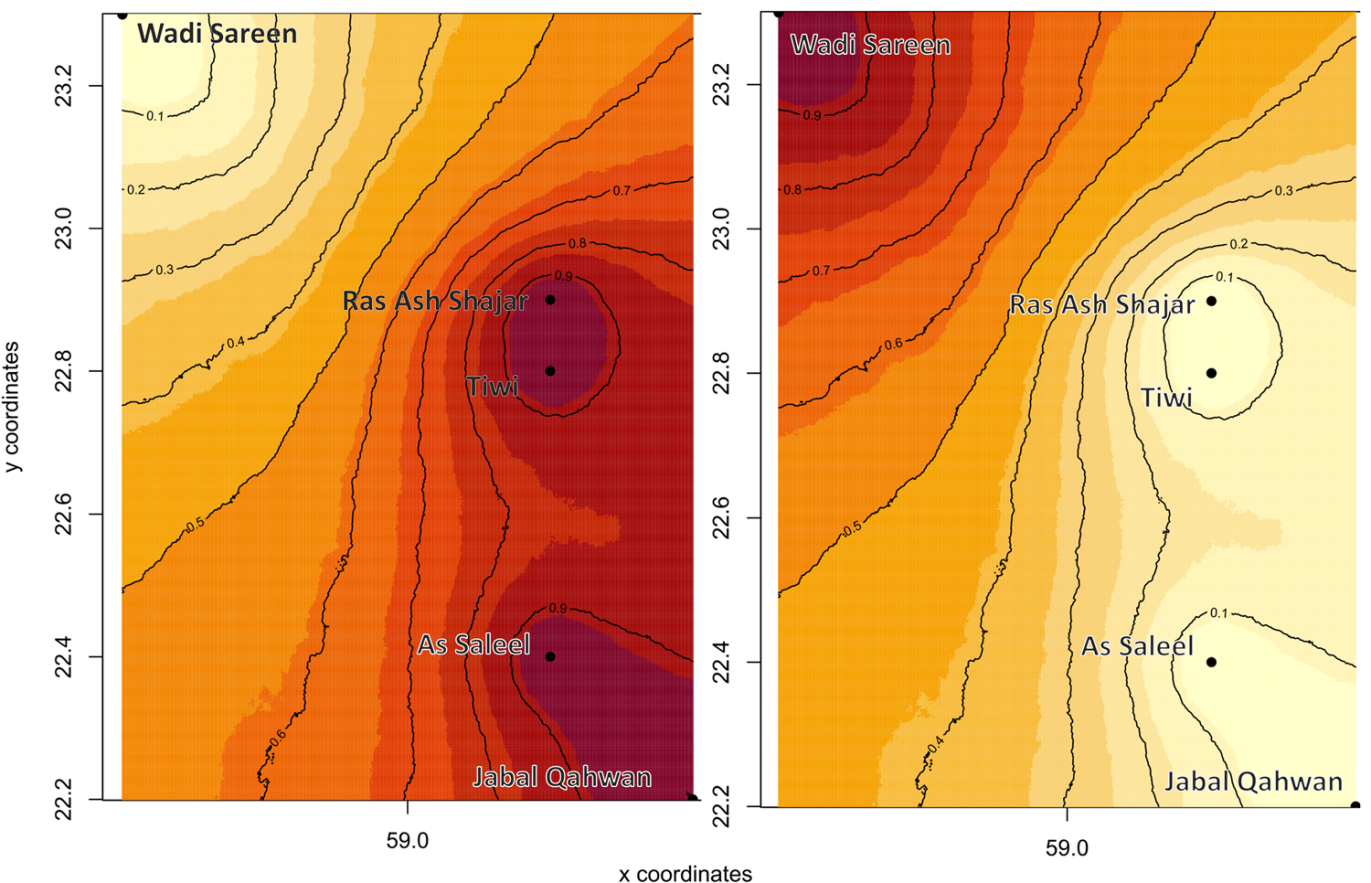
Posterior probability for the Arabian tahr to belonging to each of the two genetic clusters found by GENELAND. The darkest areas represent between 90 and 100% probability for an individual to belong to their respective genetic cluster

The results form GENELAND also showed that the *K* = 2 is the most likely scenario, however there were not a large difference between *K* = 2 and *K* = 3 (results not shown), hinting for further substructure in one of the clusters. The map of probability distribution created with GENELAND shows a similar clustering of the different sampling sites as the software STRUCTURE (Fig. 5).

**Fig. 6:**
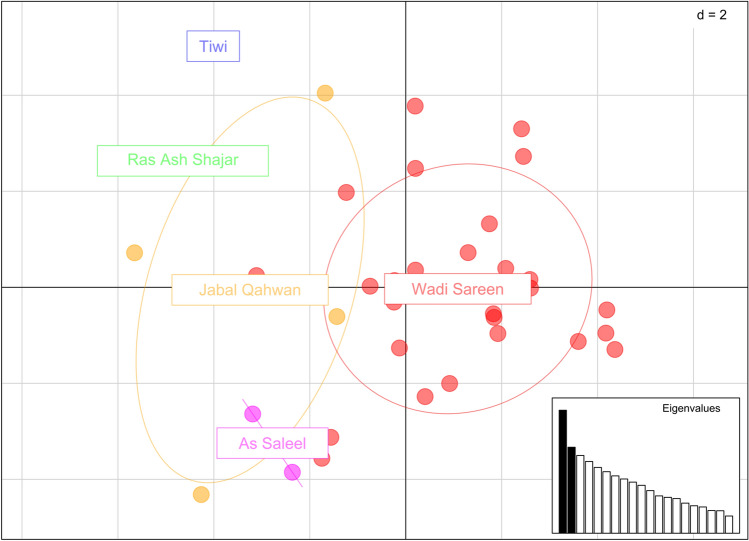
First and second component of the principle component analysis of the dataset of 14 microsatellite loci, Eigenvalue 1 = 6.73 Eigenvalue 2 = 4.71. The ellipses encompass 60% of the population’s variation

The find.cluster analysis showed no clear difference between *K* = 2 and *K* = 3 (delta BIC = 0.5) which did not allow us to estimate a precise number of genetic clusters using this method. Principle component analysis (PCA) of the data showed some degree of spatial separation (Fig. 6) between individuals from Wadi Sareen and the more southern populations which were recovered in the population assignment using Structure 2.3.4 (Fig. 4b) and in the map of posterior probability distribution using GENELAND (Fig. 5). The overlap of population clusters in the PCA also indicate the presence of admixture.

Based on this finding, for further analysis, the dataset was divided into two population clusters corresponding to Wadi Sareen and the populations south of Wadi Sareen (Tiwi, Ras Ash Shajar, Jabal Qahwan and As Saleel).

The analysis of molecular variance (AMOVA) is summarised in Table 4. This highlights that the majority of variance in microsatellite allele frequency was found within individuals relative to the entire population (69%). However, significant proportions of the variance were also attributable to the difference among individuals within populations (17%) and the difference between populations (14%).

**Table 4:** AMOVA at 14 microsatellite loci for Arabian tahr samples from Oman grouped into “Wadi Sareen” and “south of Wadi Sareen”

Variation	*df*	Sum of squares	Variance components	% Variation
Among Pops (*Wadi Sareen* versus south of *Wadi Sareen*)	1	231.673	21.673	14
Among individuals within pops	35	163.827	4.681	17
Within individuals	37	116.000	3.135	69
Total	73	3301.500		

Pairwise *F*_st_ between the two populations was 0.0651 (*P* value: 0.0079). Regarding the genetic diversity indices *H*_e_ was 0.509 ± 0.136 (s.d)—0.472 ± 0.255 (s.d) and *H*_o_ was 0.512 ± 0.161 (s.d)—0.364 ± 0.206 (s.d) for Wadi Sareen and south of Wadi Sareen respectively. The inbreeding coefficient (*F*_is_) was 0.012 (95% confidence interval (CI): − 0.088 to 0.077) for Wadi Sareen and 0.299 (CI: − 0.004 to 0.408) south of Wadi Sareen.

### ddRAD SNP data

Following filtering of 12.3 million reads, 1314 variable SNPs loci were identified across the dataset. Out of the 21 samples analysed, 19 could be used for further analysis. Mean expected heterozygosity (*H*_e_) was 0.288 ± 0.157 (s.d). Mean observed heterozygosity (*H*_o_) was 0.312 ± 0.199 (s.d). Of these variable SNPs 342 had flanking region > 50 bp either side of the SNP, making them suitable for future assay design.

Analysis of the dataset with structure (*K* = 1–3) and the find.cluster function from the adegenet package revealed no clear substructure within Wadi Sareen. However, the presence of admixed individuals could be observed (Online Appendix 5).

### Landscape connectivity

The AUC values for the five connectivity models varied from 0.62 to 0.71. The best connectivity model thus had fair ability to discriminate between tahr presence or absence at camera trap sites using predicted current flow (Pearce and Ferrier 2000). All models suggested that the Jabal Qahwan and As Saleel Nature Reserves were quite isolated from the northern reserves in the study area due to the cumulative effects of distance and resistance to movement. Only the low resistance model, where connectivity and current flow were higher, simulating times before anthropogenic developments, showed the As Saleel Nature Reserve was connected. However, even under conditions of low resistance to movement, the Jabal Qahwan and As Saleel populations were relatively poorly connected to the northern populations.

The separation between tahr populations in the north and south of the study area was supported by our genetic data (mtDNA and microsatellite), which indicated a low level of genetic exchange between the north and south. However, while genetic data suggested the split between the populations was located immediately south of Wadi Sareen, the landscape connectivity data indicated the split was south of Ras Ash Shajar and Tiwi. As a very small number of genetic samples were tested from this area, one individual from Tiwi and three from Ras Ash Shajar, it may be that the Tiwi and Ras Ash Shajar populations are genetically closer to Wadi Sareen but the few individuals we tested happened to be genetically closer to Jabal Qahwan. For this reason, we believe the split between populations is most likely south of Tiwi, isolating only the As Saleel and Jabal Qahwan populations.

**Fig. 7:**
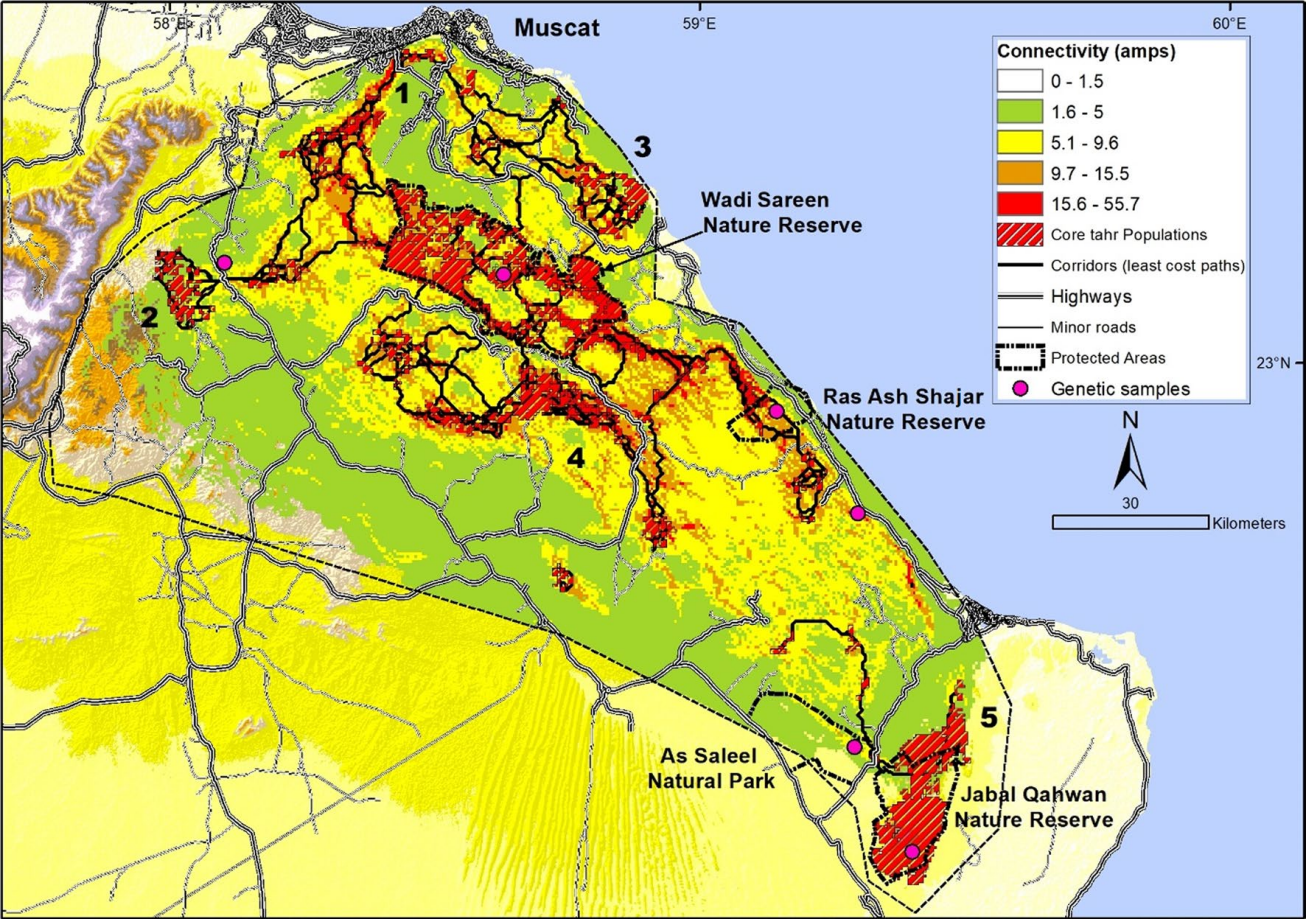
Map showing cumulative current (amps), least cost path corridors connecting core Arabian tahr populations in the study area (black dashed line) in the Hajar Mountains of Oman. The numbers (1–5) represent the different populations separated by highways, (1) Muscat, (2) Samail, (3) Satari, (4) Mehlah-Tool and (5) Jabal Qahwan

The best connectivity model indicated a total of 11 corridors impeded by highways, these impediments affected five separate tahr populations (Fig. 7). These include: (1) the Muscat population which has four highways bisecting a small tahr population located in the mountains surrounding Muscat; (2) the Samail population which may be one of the only viable corridors connecting the southern tahr populations (studied here) and the northern population (See Ross et al. 2017); (3) the Satari population which is suspected to be large and is in the process of being made into a protected area; (4) the Mehlah-Tool population which is large and has several corridors to Wadi Sareen; and (5) the Jabal Qahwan population which is the second largest single population of Arabian tahr and has recently been blocked by a new highway development (Fig. 7). All of the connections were confirmed during recognisance and the feasibility of restoration of the connections assessed. While all of the areas are important priorities, the corridors connecting Jabal Qahwan and Samail are clear conservation management priorities. Blockages to the Jabal Qahwan population seriously threatens its viability, whereas connections to Samail are important to maintain connectivity and gene flow between the southern study population and tahr populations north of the study area.

## Discussion

This study provides useful molecular and landscape recommendations and tools that improve our understanding of population structure, genetic diversity and landscape management of the Arabian tahr. These management tools and recommendations facilitate the preservation of population connectivity and the genetic integrity of tahr within the species range.

### Development of a genetic toolkit for the Arabian tahr

The use of multiple genetic methods in this study allowed comparison of the reliability of results from a number of techniques. The mitochondrial control regions (d-loop) provided important information about the genetic structure thanks to the presence of nine different haplotypes. These haplotypes presented a strong geographical segregation permitting us to define different genetic haplogroups in our dataset. Other studies on related species also successfully used mitochondrial DNA in order to identify monophyletic haplogroups (Gebremedhin et al. 2009), define the genetic distance between different populations (Joshi et al. 2018) or estimate the level of introgression between wild and domestic species (Hammer et al. 2008). Analyses using mitochondrial DNA is relatively inexpensive and the primers tested in this article could be easily use on larger datasets with specific conservation goals. Because mtDNA has the tendency to degrade at a slower rate than nuclear DNA, it is a reliable source of DNA for non-invasive samples analysis. It allows for rapid assessment of genetic diversity within and between populations across the study area. However, using only one loci does not provide a sufficient level of precision for highly admixed populations.

A better level of precision was gained from the panel of 14 microsatellite loci than from the mitochondrial DNA, in terms of understanding of genetic diversity and population structure in the study area. In addition, the probability of identity (PID) and probability of identity for siblings (PIDsibs) results showed that the microsatellite panel was informative enough to carry out individual and sibling identification. This suggests that this panel could be used to conduct mark-recapture population surveys of Arabian tahr using non-invasive genetic samples. Mark-recapture methods have been used on many other similarly elusive mammals such as the Eurasian Otter (*Lutra lutra*) (Lampa et al. 2015) and Asian elephant (*Elephas maximus*) (Gray et al. 2014). Though given the difficulty of finding Arabian tahr genetic samples, due to their low density and pellet samples often being hidden under rocks, mark-recapture may only be practical in relatively high density tahr populations. However, it is possible that the occupancy/distribution map created by Ross et al. (2017) could facilitate more targeted sample collection in areas with more likelihood of tahr occupancy.

Microsatellites are good genetic markers to use when only non-invasive samples are available. They can give a large amount of information, even when the DNA is degraded, and providing enough markers are used. However, if used with non-invasive samples they require running every sample in triplicate or more in order to decrease the chances of scoring error (Miquel et al. 2006).

Non-invasive samples can be a challenging source of DNA to work with (Taberlet et al. 1999) but are often the only source of DNA available, or best that we have access to, particularly when working with rare species (Piggott and Taylor 2003) in difficult to access areas. Based on the amount of successfully amplified samples, we demonstrated that non-invasive samples are a reliable source of DNA for monitoring Arabian tahr populations using mitochondrial DNA or microsatellite markers.

Finally the large number of SNPs developed in this study could be used to precisely assess genetic diversity and population differentiation even with a small number of individuals per population (Nazareno et al. 2017). For example, SNPs could give information regarding the development and success of translocation plans (Senn et al. 2014) or could be used to understand breeding success and family structure in the wild. Using ddRAD analysis to find SNPs is a great and relatively inexpensive way to get a large amount of data. However, ddRAD requires a high quantity and quality of DNA and therefore cannot easily be used with non-invasive samples.

### Population structure and connectivity

The results of population structure analysis (STRUCTURE, GENELAND, PCA and AMOVA) suggested two main genetic clusters in the study area, the Wadi Sareen cluster in the north and a cluster south of Wadi Sareen. Given that we would expect a time lag on the effects of barriers to gene flow, it is likely that the genetic differentiation that we observed was the result of local environmental conditions occurring 30 or more years ago (Landguth et al. 2010). As only 40 years have passed since the first notable anthropogenic fragmentation of habitat was initiated in the Hajar Mountains (Didero et al. 2019), the observed genetic structure was most likely due to natural phenomenon. Supporting this, connectivity analyses of a model with low resistance to movement and simulating historic landscapes, suggested connectivity between the northern and the As Saleel-Jabal Qahwan population was low (see Online Appendix 6). It seems likely that genetic exchange may have always been naturally low due to the cumulative effects of landscape resistance and distance between the populations. The spatially explicit clustering analysis realised with GENELAND seem to also support the connectivity analysis model. Indeed, the map of probability membership suggest a low level of genetic exchanges between the Wadi Sareen cluster and the cluster south of Wadi Sareen (Fig. 5).

Since the 1980s Oman has seen rapid development and urbanisation (Didero et al. 2019), consequently anthropogenic impacts have increased dramatically and reduced landscape connectivity. The expansion of villages and creation of multilane highways now block or restrict many historic wildlife corridors. The reduced connectivity will likely reduce genetic exchange and result in further genetic sub-structuring of tahr populations, as has been observed in other mountain ungulates such as desert bighorn sheep (*Ovis canadensis nelsoni*; (Epps et al. 2005). Our analyses highlighted numerous corridors that are experiencing issues due to anthropogenic impacts, particularly from highways. The northern Wadi Sareen population has numerous potential connections to the surrounding landscape, but the majority are impeded by highways. The situation in the southern genetic cluster is more dire. The Jabal Qahwan and As Saleel tahr populations are now quite isolated from those in the north, and a new highway running between Jabal Qahwan and As Saleel, further limits genetic exchange between these two populations.

The Wadi Sareen genetic cluster had higher heterozygosity (*H*_e_ and *H*_o_) and a lower inbreeding coefficient (*F*_is_) than the southern cluster. The Wadi Sareen population is the largest contiguous Arabian tahr population in existence and is the least threatened population in our sampling area (Ross et al. 2017). Although, Jabal Qahwan has similar density of Arabian tahr, it is approximately 2.5 times smaller in area than Wadi Sareen and thus has a smaller population size (Ross, Unpublished data). The lower population size in the southern genetic cluster may contribute to the lower observed heterozygosity and higher inbreeding coefficient when compared with the northern cluster. A small number of individuals, combined with isolation, would make the southern population more sensitive to genetic drift and would result in a lower genetic diversity (Allendorf et al. 2012). However, the presence of admixture in some individuals of Wadi Sareen indicated that some individuals do move between the two genetic clusters. We could speculate that a source sink dynamic in favour of Wadi Sareen is taking place, which would also explain the higher level of genetic diversity in this area. Some analysis of geneflow with a larger sample size would be necessary to confirm this theory.

Despite some possible exchanges between the two genetic clusters, the low genetic diversity and high *F*_is_ results of the southern genetic clusters indicate that these exchanges might be too rare to maintain a genetically sustainable population in the future. Given these concerns increasing the connectivity between Wadi Sareen and the southern sites should be prioritised to facilitate the exchange of genes to help increase genetic diversity and decrease the level of inbreeding in the southern population. Considering the likelihood of further reduction in genetic exchange between the northern and southern clusters, it would be wise to treat Jabal Qahwan—As Saleel as a separate management unit. The aims and objectives for this management unit should be geared towards maintaining connectivity with northern tahr populations.

### Conservation management of Arabian tahr

Our results give us a better understanding of connectivity between Arabian tahr in its southern range. The ecology of this rare and endemic species is still not well understood and concerted effort is required to protect remaining populations. Management actions should include improvement and protection of corridors, periodic genetic monitoring and a population viability analyses (PVA; e.g. Lacy (2000). Although restoration of habitat corridors is ongoing or if connectivity to this population proves unviable, translocation of individuals from northern populations could be used to help mitigate the effects of inbreeding depression and low genetic diversity by reinforcing the weak genepool. This method has proved successful for many other mammal species (Soorae 2018). However, as tahr populations in Oman are currently genetically viable, maintaining protected areas and preservation and restoration of connectivity between habitats should be prioritised to maintain genetic viability.

Our study highlighted highways as a threat to wildlife connectivity in the study area. A total of 11 important tahr corridors were negatively impacted by highways (Fig. 7). Connections between As Saleel and Jabal Qahwan, and between tahr populations north of the study area were particularly affected. The source population of Wadi Sareen Nature Reserve was also impacted, with most corridors out of Wadi Sareen impeded by highways. A solution is required to mitigate the impact of highways on wildlife movement in Oman. As all main roads in Oman, and particularly in the mountains, are equipped with frequent road underpasses/culverts to protect the road against flash floods, one potential option is to use these existing culverts as wildlife corridors. The culverts are generally from 3 to 10 m wide and in theory provide suitable wildlife crossings under roads. The majority of culverts are located in Wadi (dry riverbed) habitats which also provide good habitat and cover for wildlife movement. In high density Arabian gazelle (*Gazella arabica*) populations in Oman culverts are known to be used by gazelle (Authors, Unpublished data), although we currently have no evidence that Arabian tahr use culverts, in the right habitats it is likely that they do. Culverts may therefore help mitigate the issue of fragmentation caused by highways, however, some steps are required to facilitate the use of culverts as corridors. These include: (1) initiating research to understand the use of culverts by wildlife in Oman and identify habitat characteristics of preferred crossing areas; (2) protection of habitat surrounding important culvert crossings for connectivity; (3) remediation and provision of corridor habitats to connect occupied tahr habitats and provide essential cover habitat, through planting trees, providing large rocks and other cover to encourage wildlife utilisation.

The preservation and restoration of Arabian tahr movement and connectivity between Oman’s protected area network and important populations is vital for maintaining genetic diversity. Human activities have restricted movement of Arabian tahr in the Hajar Mountains and are most likely contributing towards reduced gene flow. In light of our results, research and actions are required to understand and restore functional connectivity to assure the long-term persistence of Arabian tahr and other wildlife species in Oman.

## Electronic supplementary material

Below is the link to the electronic supplementary material.

Supplementary file1 (XLSX 10 kb)

Supplementary file2 (XLSX 11 kb)

Supplementary file3 (XLSX 9 kb)

Supplementary file4 (PNG 210 kb)

Supplementary file5 (PNG 641 kb)

Supplementary file6 (PDF 1319 kb)

Supplementary file7 (PDF 111 kb)

## Data Availability

The mitochondrial control region sequences are available on GenBank with accession numbers: MN563184, MN563185, MN563186, MN563187, MN563188, MN563189, MN563190 and MN563191. The results of the microsatellite genotyping, the SNPs loci file, the script used for the PCA analysis, the GIS files, the landscape resistance values and the camera trap data are available on the figshare repository: Genetic analysis data: 10.6084/m9.figshare.12905984. Landscape analysis data: 10.6084/m9.figshare.12896975. Information about the samples used in this study has been deposited in the CryoArks database https://www.cryoarks.org/.

## References

[CR1] Adriaensen F, Chardon JP, De Blust G (2003). The application of ‘least-cost’modelling as a functional landscape model. Landsc Urban Plan.

[CR2] Allendorf FW, Luikart GH, Aitken S (2012). Conservation and the genetics of populations.

[CR3] Baden AL, Mancini AN, Federman S (2019). Anthropogenic pressures drive population genetic structuring across a critically endangered lemur species range. Sci Rep.

[CR4] Beier P, Noss RF (1998). Do habitat corridors provide connectivity?. Conserv Biol.

[CR5] Belkhir K, Borsa P, Chikhi L et al (2004) GENETIX 4.05, logiciel sous Windows TM pour la génétique des populations. Laboratoire Génome, Populations, Interactions, CNRS UMR 5171, Université de Montpellier II, Montpellier (France)

[CR6] Bourgeois S, Senn H, Kaden J (2018). Single-nucleotide polymorphism discovery and panel characterization in the African forest elephant. Ecol Evol.

[CR7] Catchen J, Hohenlohe PA, Bassham S (2013). Stacks: an analysis tool set for population genomics. Mol Ecol.

[CR8] Didero M, Farooq A, Nebel S, Pfaffenbach C (2019). Urban Oman: from modern to postmodern mobility in Muscat?. Meta.

[CR9] Dutta T, Sharma S, McRae BH (2016). Connecting the dots: mapping habitat connectivity for tigers in central India. Reg Environ Change.

[CR10] Earl DA, vonHoldt BM (2012). STRUCTURE HARVESTER: a website and program for visualizing STRUCTURE output and implementing the Evanno method. Conserv Genet Resour.

[CR11] Eng J (2014) ROC analysis: web-based calculator for ROC curves. In: Balt. Johns Hopkins Univ. https://www.jrocfit.org. Accessed 5 Dec 2019

[CR12] Epps CW, Palsbøll PJ, Wehausen JD (2005). Highways block gene flow and cause a rapid decline in genetic diversity of desert bighorn sheep. Ecol Lett.

[CR13] ESRI (2015) Redlands CA, ArcMap 10.3.1

[CR14] Evanno G, Regnaut S, Goudet J (2005). Detecting the number of clusters of individuals using the software STRUCTURE: a simulation study. Mol Ecol.

[CR15] Excoffier L, Lischer HEL (2010). Arlequin suite ver 3.5: a new series of programs to perform population genetics analyses under Linux and Windows. Mol Ecol Resour.

[CR16] Fahrig L (2003). Effects of habitat fragmentation on biodiversity. Annu Rev Ecol Evol Syst.

[CR17] Frankham R (2015). Genetic rescue of small inbred populations: meta-analysis reveals large and consistent benefits of gene flow. Mol Ecol.

[CR18] Frankham R, Bradshaw CJA, Brook BW (2014). Genetics in conservation management: revised recommendations for the 50/500 rules, red list criteria and population viability analyses. Biol Conserv.

[CR19] Gebremedhin B, Ficetola GF, Naderi S (2009). Combining genetic and ecological data to assess the conservation status of the endangered Ethiopian walia ibex. Anim Conserv.

[CR20] Gray TNE, Vidya TNC, Potdar S (2014). Population size estimation of an Asian elephant population in eastern Cambodia through non-invasive mark-recapture sampling. Conserv Genet.

[CR21] Hammer SE, Schwammer HM, Suchentrunk F (2008). Evidence for introgressive hybridization of captive markhor (*Capra falconeri*) with domestic goat: cautions for reintroduction. Biochem Genet.

[CR22] Hassanin A, Ropiquet A, Couloux A, Cruaud C (2009). Evolution of the mitochondrial genome in mammals living at high altitude: new insights from a study of the tribe Caprini (Bovidae, Antilopinae). J Mol Evol.

[CR23] Hubisz MJ, Falush D, Stephens M, Pritchard JK (2009). Inferring weak population structure with the assistance of sample group information. Mol Ecol Resour.

[CR24] Jombart T (2008). adegenet: a R package for the multivariate analysis of genetic markers. Bioinformatics.

[CR25] Jombart T, Ahmed I (2011). Adegenet 1.3-1: new tools for the analysis of genome-wide SNP data. Bioinformatics.

[CR26] Joshi BD, Matura R, Predit MA (2018). Palghat gap reveals presence of two diverged populations of Nilgiri tahr (*Nilgiritragus hylocrius*) in Western Ghats, India. Mitochondrial DNA Part B.

[CR27] Kopelman NM, Mayzel J, Jakobsson M (2015). Clumpak: a program for identifying clustering modes and packaging population structure inferences across K. Mol Ecol Resour.

[CR28] Lacy RC (2000). Structure of the VORTEX simulation model for population viability analysis. Ecol Bull.

[CR29] Lampa S, Mihoub JB, Gruber B (2015). Non-invasive genetic mark-recapture as a means to study population sizes and marking behaviour of the elusive Eurasian otter (*Lutra lutra*). PLoS ONE.

[CR30] Landguth EL, Cushman SA, Schwartz MK (2010). Quantifying the lag time to detect barriers in landscape genetics. Mol Ecol.

[CR31] Leigh JW, Bryant D (2015). POPART: full-feature software for haplotype network construction. Methods Ecol Evol.

[CR32] McRae BH, Kavanagh DM (2011). Linkage mapper connectivity analysis software.

[CR33] Mcrae BH, Dickson BG, Keitt TH (2008). Using circuit theory to model connectivity in ecology, evolution, and conservation. Ecology.

[CR34] McRae BH, Shah VB, Mohapatra TK (2013). Circuitscape 4 user guide.

[CR35] McRae BH, Shirk A, Platt J (2013). Gnarly landscape utilities: resistance and habitat calculator user guide.

[CR36] Miquel C, Bellemain E, Poillot C (2006). Quality indexes to assess the reliability of genotypes in studies using noninvasive sampling and multiple-tube approach. Mol Ecol Notes.

[CR37] Nazareno AG, Bemmels JB, Dick CW, Lohmann LG (2017). Minimum sample sizes for population genomics: an empirical study from an Amazonian plant species. Mol Ecol Resour.

[CR38] Noss R (2004) Can urban areas have ecological integrity. In: Shaw W, Harris L, VanDruff L (eds) Proceedings, 4th Int Wildl Symp, pp 3–8

[CR39] Peakall R, Smouse PE (2012). GenALEx 6.5: genetic analysis in Excel. Population genetic software for teaching and research-an update. Bioinformatics.

[CR40] Pearce J, Ferrier S (2000). Evaluating the predictive performance of habitat models developed using logistic regression. Ecol Modell.

[CR41] Peterson BK, Weber JN, Kay EH (2012). Double digest RADseq: an inexpensive method for de novo SNP discovery and genotyping in model and non-model species. PLoS ONE.

[CR42] Piggott MP, Taylor AC (2003). Remote collection of animal DNA and its applications in conservation management and understanding the population biology of rare and cryptic species. Wildl Res.

[CR43] Pritchard JK, Stephens M, Donnelly P (2000). Inference of population structure using multilocus genotype data. Genetics.

[CR44] Purcell S, Neale B, Todd-Brown K (2007). PLINK: a tool set for whole-genome association and population-based linkage analyses. Am J Hum Genet.

[CR45] Raymond M, Rousset F (1995). GENEPOP (Version 1.2): population genetics software for exact tests and ecumenicism. J Hered.

[CR46] Reed DH, Frankham R (2003). Society for conservation biology correlation between fitness and genetic diversity. Conserv Biol.

[CR47] Ropiquet A, Hassanin A (2005). Molecular evidence for the polyphyly of the genus *Hemitragus* (Mammalia, Bovidae). Mol Phylogenet Evol.

[CR48] Ross S, Al Jahdhami MH, Al Rawahi H (2017). Refining conservation strategies using distribution modelling: a case study of the endangered Arabian tahr *Arabitragus jayakari*. Oryx.

[CR49] Ross S, Al-Rawahi H, Al-Jahdhami MH, et al (2019) Arabitragus jayakari. In: The IUCN Red List of Threatened Species 2019: e.T9918A128770408. 10.2305/IUCN.UK.2019-1.RLTS.T9918A128770408.en. Accessed 1 Jul 2019

[CR50] Sala OE, Chapin FS, Armesto JJ (2000). Global biodiversity scenarios for the year 2100. Science.

[CR51] Senn H, Ogden R, Frosch C (2014). Nuclear and mitochondrial genetic structure in the Eurasian beaver (Castor fiber)—implications for future reintroductions. Evol Appl.

[CR52] Soorae PS (2018) Global reintroduction perspectives: 2018. Case studies from around the globe. IUCN, International Union for Conservation of Nature, Gland, Switzerland : IUCN SSC Reintroduction Specialist Group and Abu Dhabi, AE : Environment Agency-Abu Dhabi

[CR53] Taberlet P, Waits LP, Luikart G (1999). Noninvasive genetic sampling look before you leap. Trends Ecol Evol.

[CR54] Van Oosterhout C, Hutchinson W, Wills D, Shipley P (2004). MICRO-CHECKER: Software for identifying and correcting genotyping errors in microsatellite data. Mol Ecol Notes.

[CR55] Verma SK, Singh L (2003). Novel universal primers establish identity of an enormous number of animal species for forensic application. Mol Ecol Notes.

[CR56] Werhahn G, Senn H, Kaden J (2017). Phylogenetic evidence for the ancient himalayan wolf: towards a clarification of its taxonomic status based on genetic sampling from Western Nepal. R Soc Open Sci.

