# Supplementary figures and images for: First evaluation of the population structure, genetic diversity and landscape connectivity of the Endangered Arabian tahr
Source: Mamm Biol. 2020 Oct 13;100(6):659–73. doi: 10.1007/s42991-020-00072-4 (PMC7661410; doi:10.1007/s42991-020-00072-4)

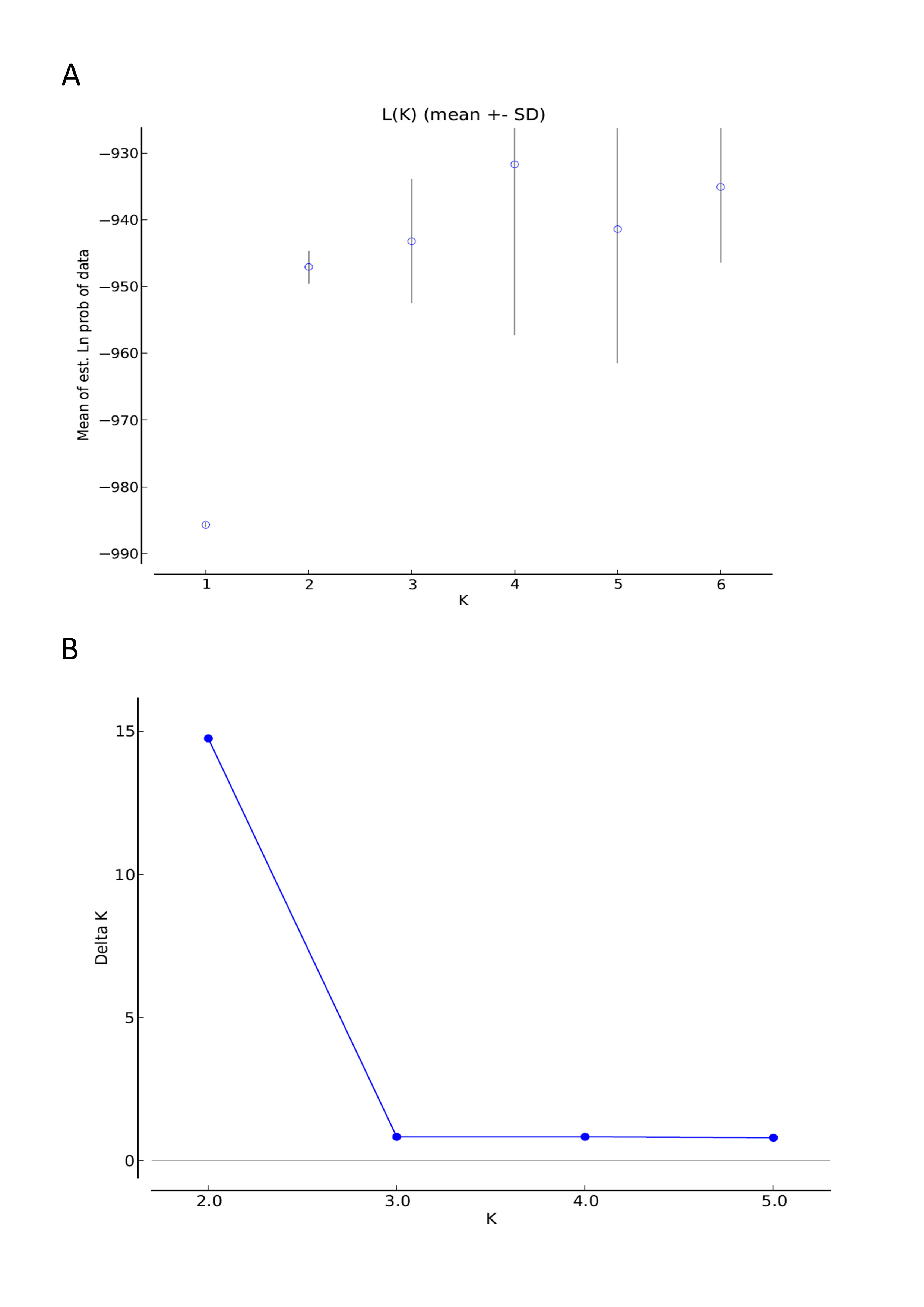

Supplement: Supplementary file 4 — Supplementary file4 (PNG 210 kb) [file 42991_2020_72_MOESM4_ESM.png]

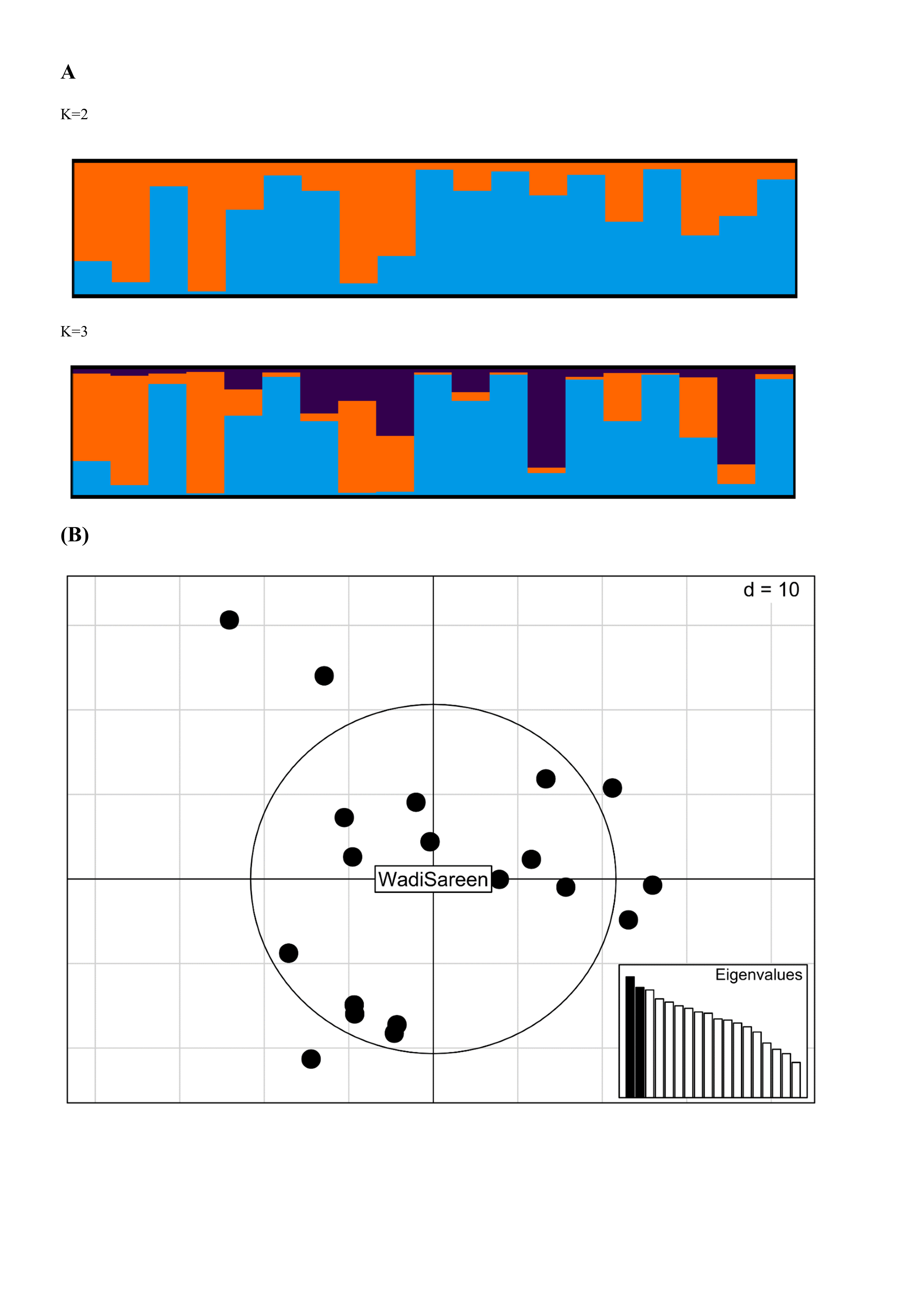

Supplement: Supplementary file 5 — Supplementary file5 (PNG 641 kb) [file 42991_2020_72_MOESM5_ESM.png]
