# Supplementary material for: First evaluation of the population structure, genetic diversity and landscape connectivity of the Endangered Arabian tahr
Source: Mamm Biol. 2020 Oct 13;100(6):659–73. doi: 10.1007/s42991-020-00072-4 (PMC7661410; doi:10.1007/s42991-020-00072-4)
Supplement: Supplementary file 6 — Supplementary file6 (PDF 1319 kb) [file 42991_2020_72_MOESM6_ESM.pdf]

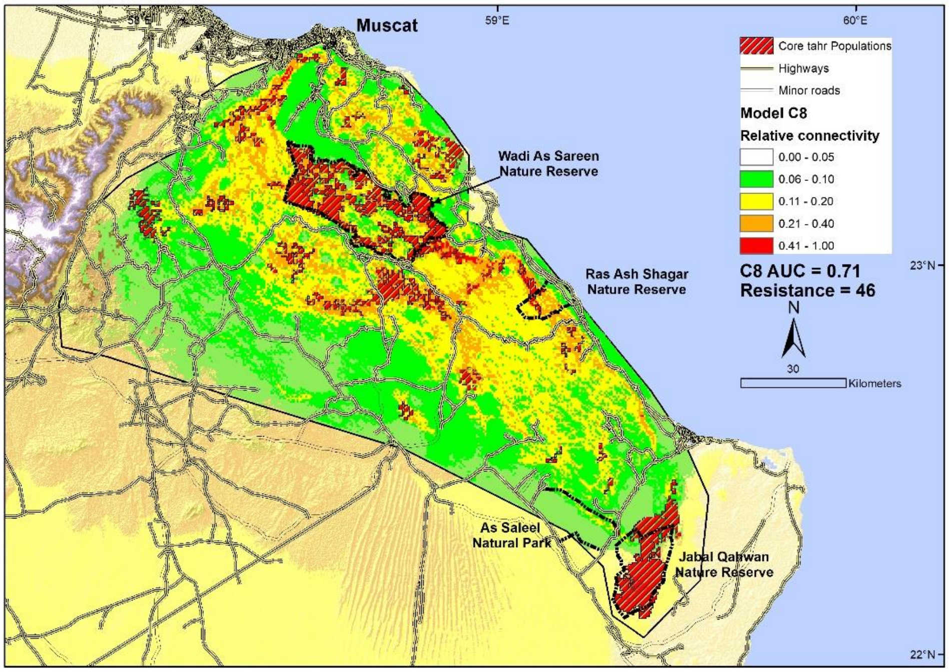

The best model according to ROC. AUC = 0.71. Total resistance = 46.

C8 Resistance Values

Data Layer	Class ID	Range	Description	Resistance	Cells
Minor Road	1	all		6	0
Major Road	1	all		11	0
Village	1	all		11	3
Ruggedness	1	1 to 1.02	High res	5	0
Ruggedness	2	1.02 to 1.05	Can travel	2	0
Ruggedness	3	1.05 to 1.2	Good	0	0
Ruggedness	4	> 1.2	Excellent	0	0
Tahr Habitat	1	0	Unused	6	0
Tahr Habitat	2	1	Rarely use	3	0
Tahr Habitat	3	2 to 3	Marginal	2	0
Tahr Habitat	4	4 to 5	Good	0	0
Tahr Habitat	5	6 to 10	Excellent	0	0

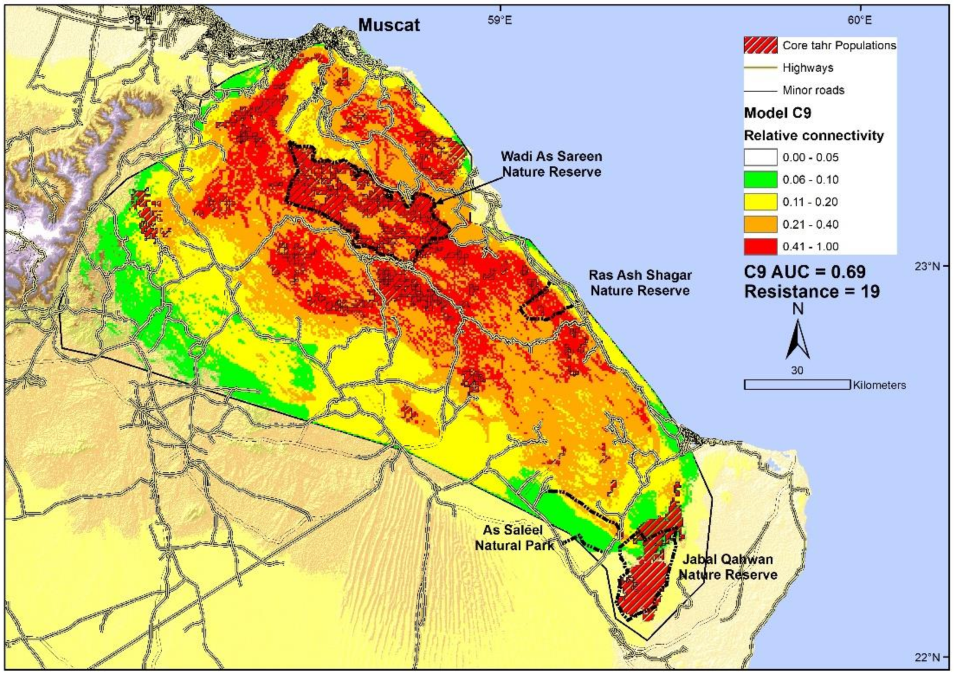

The second-best model according to ROC. AUC = 0.69. Total resistance = 19.

C9 resistance values

Data Layer	Class ID	Range	Description	Resistance	Cells
Minor Road	1	all		3	0
Major Road	1	all		5	0
Village	1	all		5	0
Ruggedness	1	1 to 1.02	High res	2	0
Ruggedness	2	1.02 to 1.05	Can travel	1	0
Ruggedness	3	1.05 to 1.2	Good	0	0
Ruggedness	4	> 1.2	Excellent	0	0
Tahr Habitat	1	0	Unused	2	0
Tahr Habitat	2	1	Rarely use	1	0
Tahr Habitat	3	2 to 3	Marginal	0	0
Tahr Habitat	4	4 to 5	Good	0	0
Tahr Habitat	5	6 to 10	Excellent	0	0

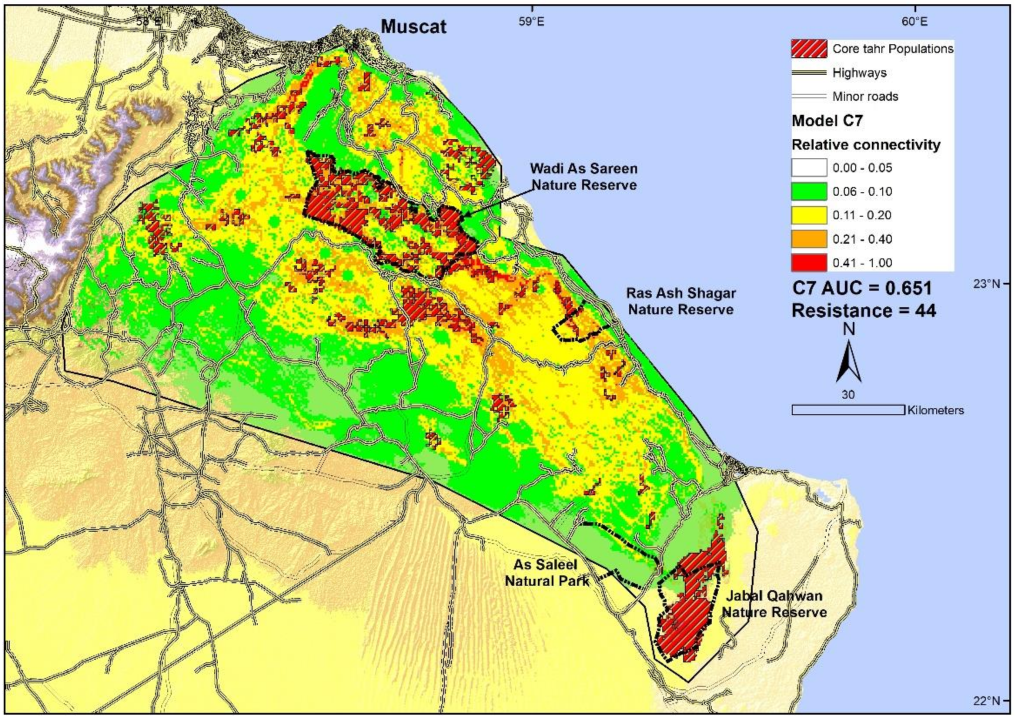

The third best model according to ROC. AUC = 0.65. Total resistance = 44.

C7 resistance values

Data Layer	Class ID	Range	Description	Resistance	Cells
Minor Road	1	all		6	0
Major Road	1	all		10	0
Village	1	all		11	3
Ruggedness	1	1 to 1.02	High res	5	0
Ruggedness	2	1.02 to 1.05	Can travel	2	0
Ruggedness	3	1.05 to 1.2	Good	0	0
Ruggedness	4	> 1.2	Excellent	0	0
Tahr Habitat	1	0	Unused	5	0
Tahr Habitat	2	1	Rarely use	3	0
Tahr Habitat	3	2 to 3	Marginal	2	0
Tahr Habitat	4	4 to 5	Good	0	0
Tahr Habitat	5	6 to 10	Excellent	0	0

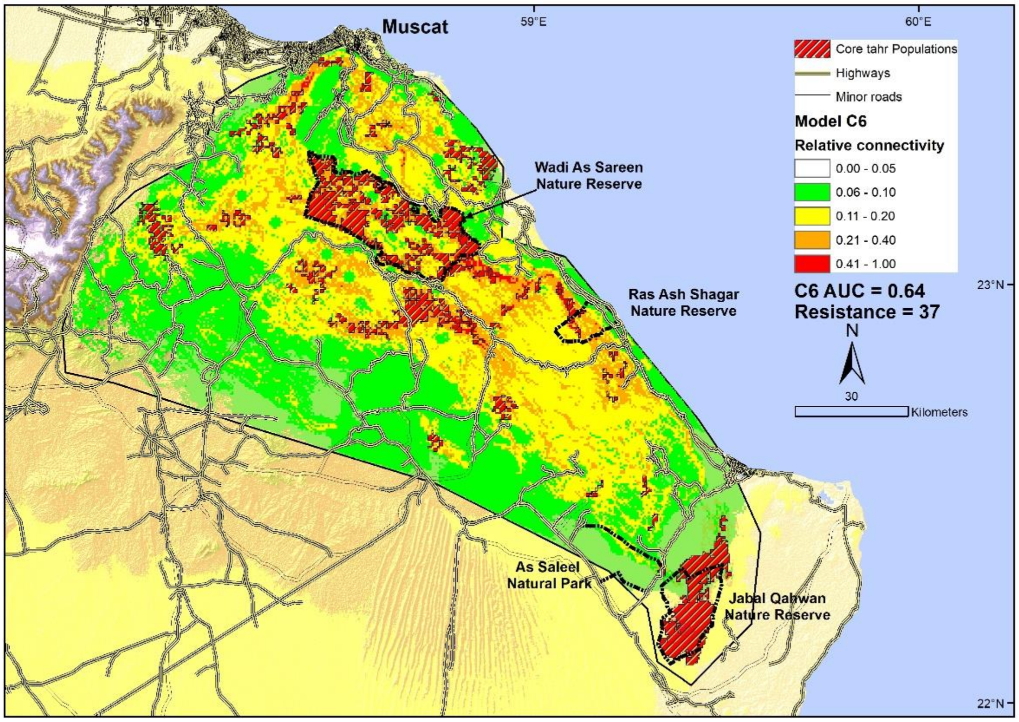

The fourth best model according to ROC. AUC = 0.64. Total resistance = 37.

C6 resistance values

Data Layer	Class ID	Range	Description	Resistance	ExpandCells
Minor Road	1	all		4	0
Major Road	1	all		8	0
Village	1	all		10	3
Ruggedness	1	1 to 1.02	High res	4	0
Ruggedness	2	1.02 to 1.05	Can travel	2	0
Ruggedness	3	1.05 to 1.2	Good	0	0
Ruggedness	4	> 1.2	Excellent	0	0
Tahr Habitat	1	0	Unused	4	0
Tahr Habitat	2	1	Rarely use	3	0
Tahr Habitat	3	2 to 3	Marginal	2	0
Tahr Habitat	4	4 to 5	Good	0	0
Tahr Habitat	5	6 to 10	Excellent	0	0

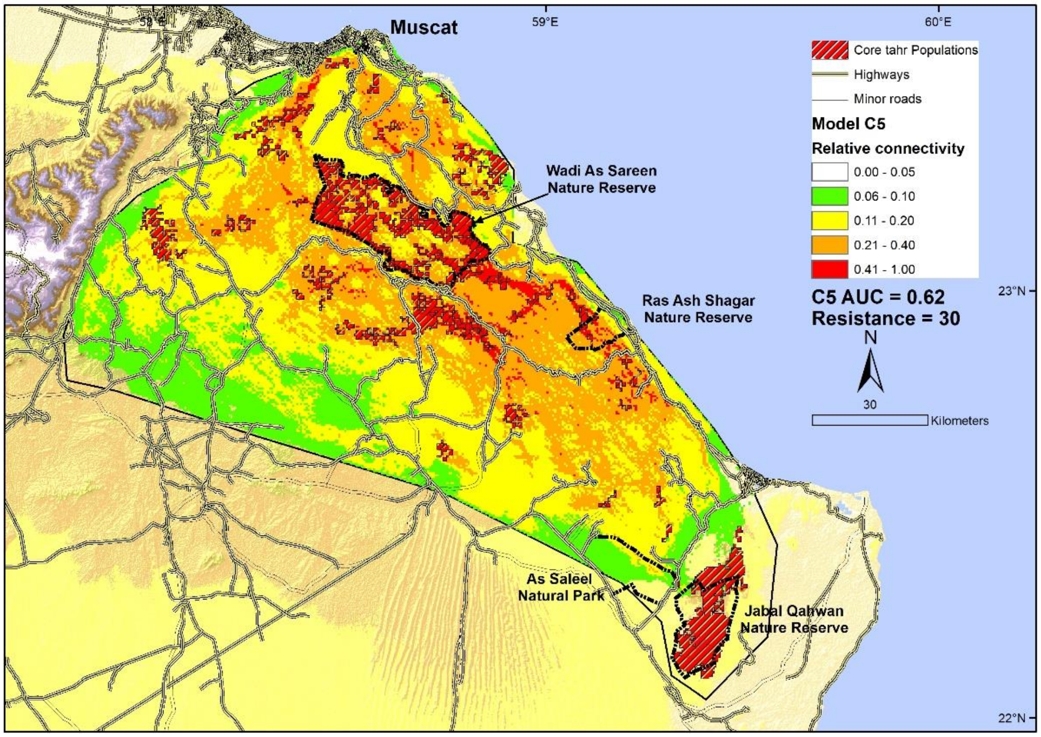

The fifth best model according to ROC. AUC = 0.62. Total resistance = 30.

C5 resistance values

Data Layer	Class ID	Range	Description	Resistance	Cells
Minor Road	1	all		3	0
Major Road	1	all		6	0
Village	1	all		8	3
Ruggedness	1	1 to 1.02	High res	4	0
Ruggedness	2	1.02 to 1.05	Can travel	2	0
Ruggedness	3	1.05 to 1.2	Good	0	0
Ruggedness	4	> 1.2	Excellent	0	0
Tahr Habitat	1	0	Unused	4	0
Tahr Habitat	2	1	Rarely use	2	0
Tahr Habitat	3	2 to 3	Marginal	1	0
Tahr Habitat	4	4 to 5	Good	0	0
Tahr Habitat	5	6 to 10	Excellent	0	0
