# Supplementary material for: First evaluation of the population structure, genetic diversity and landscape connectivity of the Endangered Arabian tahr
Source: Mamm Biol. 2020 Oct 13;100(6):659–73. doi: 10.1007/s42991-020-00072-4 (PMC7661410; doi:10.1007/s42991-020-00072-4)
Supplement: Supplementary file 7 — Supplementary file7 (PDF 111 kb) [file 42991_2020_72_MOESM7_ESM.pdf]

First evaluation of the population structure, genetic diversity and landscape connectivity of the Endangered Arabian tahr

Appendix References:

Barendse W, Armitage SM, Kossarek LM, et al (1994) A genetic linkage map of the bovine genome. Nat Genet 6:227–235

Beja-Pereira A, Zeyl E, Ouragh L, et al (2004) Twenty polymorphic microsatellites in two of North Africa's most threatened ungulates: Gazella dorcas and Ammotragus lervia (Bovidae; Artiodactyla). Mol Ecol Notes 4:452–455. https://doi.org/10.1111/j.1471-8286.2004.00678.x

Bhebhe E, Kogi J, Holder DA, et al (1994) Caprine microsatellite dinucleotide repeat polymorphisms at the SR-CRSP-6, SR-CRSP-7, SR-CRSP-8, SR-CRSP-9 and SR-CRSP-10 loci. Anim Genet 25:203

Bishop MD, Kappes SM, Keele JW, et al (1994) A Genetic Linkage Map for Cattlet Ruedi Fries. Genetics 619–639. https://doi.org/10.1039/c1em10059a

Buchanan FC, Crawford AM (1993) Ovine microsatellites at the OARFCB11, OARFCB128, OARFCB193, OARFCB266 and OARFCB304 loci. Anim Genet 24:145

Burns BM, Taylor JF, Herring KL, et al (1995) Bovine microsatellite mononucleotide and dinucleotide repeat polymorphisms at the TEXAN6, TEXAN7, TEXAN8, TEXAN9 and TEXAN10 loci. Anim Genet 26:128–129

Crawford AM, Dodds KG, Ede AJ, et al (1995) An autosomal genetic linkage map of the sheep genome. Genetics 140:703–724

Ede AJ, Pierson CA, Crawford AM (1995) Ovine microsatellites at the OarCP9, OarCP16, OarCP20, OarCP21, OarCP23, and OarCP26 loci. Anim Genet 26:129–130

Georges M, Massey JM (1992) Polymorphic DNA markers in Bovidae

Hawkins GA, Toldo SS, Bishop MD, et al (1995) Physical and linkage mapping of the bovine genome

with cosmids. Mamm Genome 6:249–254

Hoffmann I, Marsan PA, Barker JSF, et al (2004) New MoDAD marker sets to be used in diversity studies for the major farm animal species: Recommendations of a joint ISAG/FAO working group. In: Proceedings of the 29th International Conference on Animal Genetics, Meiji University. Tokyo, Japan, p 107

Huebinger RM, Pierson DJ, De Maar TW, et al (2002) Characterization of 16 microsatellite marker loci in the Maasai giraffe (Giraffa camelopardalis tippelskirchi). Mol Ecol Notes 2:531–533

Hulme DJ, Silk JP, Redwin JM, et al (1994) Ten polymorphic ovine microsatellites. Anim Genet 25:434–435

Kossarek LM, Grosse WM, Finlay O, et al (1995) Six bovine dinucleotide repeat polymorphisms: RM041, RM051, RM066, RM088, RM103 and RM113. Anim Genet 26:55–56

Machugh DE, Shriver MD, Loftus RT, et al (1997) hDomestication and Phylogeography of Taurine and Zebu Cattle

Maddox JF, Davies KP, Crawford AM, et al (2001) An enhanced linkage map of the sheep genome comprising more than 1000 loci. Genome Res 11:1275–1289. https://doi.org/10.1101/gr.GR-1350R

Penty JM, Henry HM, Ede AJ, Crawford AM (1993) Ovine microsatellites at the OarAEl6, OarAE54, OarAE57, OarAE119 and OarAE129 loci. Anim Genet 24:219

Swarbrick PA, Dietz AB, Womack JE, Crawford AM (1992a) Ovine and bovine dinucleotide repeat polymorphism at the MAF46 locus. Anim Genet 23:182

Swarbrick PA, Howes J, Crawford AM (1992b) Ovine dinucleotide repeat polymorphism at the MAF50 locus. Anim Genet 23:

Toldo SS, Fries R, Steffen P, et al (1993) Physically mapped, cosmid-derived microsatellite markers as

anchor loci on bovine chromosomes. Mamm Genome 4:720–727

Vaiman D, Mercier D, Moazami-Goudarzi K, et al (1994) A set of 99 cattle microsatellites: characterization, synteny mapping, and polymorphism. Mamm Genome 5:288–297

Wilson GA, Strobeck C, Wu L, Coffin JW (1997) Characterization of microsatellite loci in caribou Rangifer tarandus, and their use in other artiodactyls. Mol Ecol 6:697–699
